# P-1144. Injury Patterns and Infectious Complications after Battlefield-Related Burn Injuries

**DOI:** 10.1093/ofid/ofaf695.1338

**Published:** 2026-01-11

**Authors:** David R Drysdale, Matthew Geringer, Connor Wakefield, Laveta Stewart, M Leigh Carson, Leopoldo Cancio, Dan Lu, Katrin Mende, Jennifer Gurney, David R Tribble, John Kiley

**Affiliations:** Brooke Army Medical Center, San Antonio, TX; Brooke Army Medical Center, San Antonio, TX; Mike O'Callaghan Military Medical Center, Las Vegas, Nevada; Infectious Disease Clinical Research Program, Henry Jackson Foundation, Bethesda, Maryland; Uniformed Services University of the Health Sciences & Henry M. Jackson Foundation for the Advancement of Military Medicine, Bethesda, Maryland; U.S. Army Institute of Surgical Research, San Antonio, Texas; Uniformed Services University of the Health Sciences & Henry M. Jackson Foundation for the Advancement of Military Medicine, Bethesda, Maryland; Infectious Disease Clincial Research Program, JBSA Ft Sam Houston, Texas; U.S. Army Institute of Surgical Research, San Antonio, Texas; Uniformed Services University of the Health Sciences, Bethesda, Maryland; BAMC, San Antonio, Texas

## Abstract

**Background:**

Thermal injuries disrupt both the innate and adaptive immune systems, resulting in an immunocompromised state that makes infection a frequent complication. Examination of military personnel with battlefield-related burns revealed that 18% developed ≥ 1 infection and those with greater total body surface area burned (TBSA) had more infections. Here, we examine infections with regard to burn injury pattern and severity.

**Figure:**
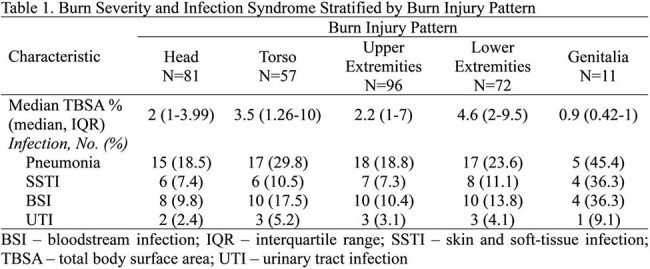

**Methods:**

Data were collected through the Trauma Infectious Disease Outcomes Study (TIDOS), an observational study of infections in U.S. military personnel who sustained deployment-related injuries (2009–2014). Patients who sustained burn injuries and were admitted to the U.S. Army Institute of Surgical Research Burn Center at Brooke Army Medical Center were included. Patients with incomplete injury pattern data were excluded. Burn injury patterns were grouped and evaluated by infection syndromes.

**Results:**

Among 144 burn patients, 136 (94%) were included in the analysis. The patients were primarily male (N=135, 99%) with combat injuries (N=83, 61%) sustained via a blast (N=76, 56%), resulting in critical injury severity (median injury severity score: 30; interquartile range: 27-41.5). There was a total of 317 burn injuries with upper extremity burns being most frequent (30%), followed by head (26%) and lower extremity burns (23%; Table 1). Lower extremity and torso burns had the greatest TBSA (% median 4.6 and 3.5, respectively). For each burn injury, pneumonia accounted for the highest proportion of infections (occurrence ranged 18.5%-45.4% per injury), followed by bloodstream infections (BSI, 9.8%-36.3%). Four patients had severe burn injury patterns (TBSA of the affected area ≥ 20%), with three having torso burns and one having lower extremities burns. All four patients with severe burns developed pneumonia.

**Conclusion:**

While the majority of burns in this study were not severe by TBSA, those that were went on to develop ≥ 1 infection, and patients with torso and genitalia burns had more pneumonia, skin and soft-tissue infections, and BSI. Understanding burn injury patterns and risk for infectious complications is critical for burn injury management and developing potential strategies for preventing infections.

**Disclosures:**

All Authors: No reported disclosures

